# Enlarged mediastinal air cyst in a patient with bronchial diverticula localized in the left main bronchus: a case report with surgical and bronchoscopic findings

**DOI:** 10.1186/s40792-016-0282-y

**Published:** 2017-01-03

**Authors:** Hideo Ichimura, Yuichiro Ozawa, Masanari Shiigai, Seiji Shiotani, Kazunori Kikuchi, Yukio Sato

**Affiliations:** 1 Department of Thoracic Surgery, Faculty of Medicine, University of Tsukuba, 2-1-1 Jyounan, Hitachi, Ibaraki 317-0077 Japan; 2 Department of Thoracic Surgery, Hitachi General Hospital, Hitachi, Japan; 3 Department of Thoracic Surgery, Tsukuba Medical Center Hospital, Tsukuba, Japan; 4 Department of Radiology, Tsukuba Medical Center Hospital, Tsukuba, Japan; 5 Department of Pathology, Tsukuba Medical Center Hospital, Tsukuba, Japan

**Keywords:** Bronchial diverticula, Mediastinal air cyst, Upper lobe-dominant pulmonary fibrosis, Surgery

## Abstract

**Background:**

A mediastinal air cyst is a rare computed tomography (CT) finding. Once the lesion is identified, it is difficult to diagnose and treat. Meanwhile, bronchial diverticula have been reported as a CT finding observed in certain pulmonary pathologic conditions. We encountered the case of an enlarged mediastinal air cyst accompanied with bronchial diverticula and upper lobe-dominant fibrous changes of the lung.

**Case presentation:**

A 69-year-old man with a chronic cough who had regularly visited a chest physician for upper lobe-dominant pulmonary fibrosis was referred to our hospital for the examination of an enlarged mediastinal air cyst. Chest CT exhibited an air cyst (size, 30 mm) connected to the lumen of the left main bronchus (LMB) and multiple tiny outpouches only on the LMB. Flexible bronchoscopy showed bubbling from slits or indentations of the bronchial mucosa only in the LMB but not in the right main bronchus or lobar bronchus. For therapeutic diagnosis, we removed the air cyst. Based on clinical, surgical, and pathological findings, we diagnosed the air cyst as an enlarged bronchial diverticulum.

**Conclusions:**

This is the first case wherein bronchoscopic and surgical findings of bronchial diverticula and an enlarged bronchial diverticulum are reported. There are possible pathogenic mechanisms in cases of pulmonary disease that are attributable to enlargement of the bronchial diverticula.

**Electronic supplementary material:**

The online version of this article (doi:10.1186/s40792-016-0282-y) contains supplementary material, which is available to authorized users.

## Background

When we incidentally detect an air cyst in the mediastinum, the lesion is left intact and monitored initially since it is presumed to be benign in nature. However, if the lesion exhibits an enlargement over time or if the patient develops symptoms, we proceed to diagnose the lesion and begin the treatment process. These processes may be challenging since mediastinal air cysts rarely develop. Conversely, the detection of bronchial diverticula (BDs), and particularly those that are less than 5 mm in size, has increased with the recent development of multi-detector row computed tomography (MDCT) [1, 2]. BDs are also rare findings and are not usually a pathologic condition; however, in some cases, therapeutic intervention may be considered. We present herein a case of an enlarged mediastinal air cyst whose CT simultaneously showed BDs in only the left main bronchus (LMB) as well as upper lobe-dominant pulmonary fibrosis. We performed surgery for therapeutic diagnosis of an enlarged air cyst.

## Case presentation

**Fig. 1:**
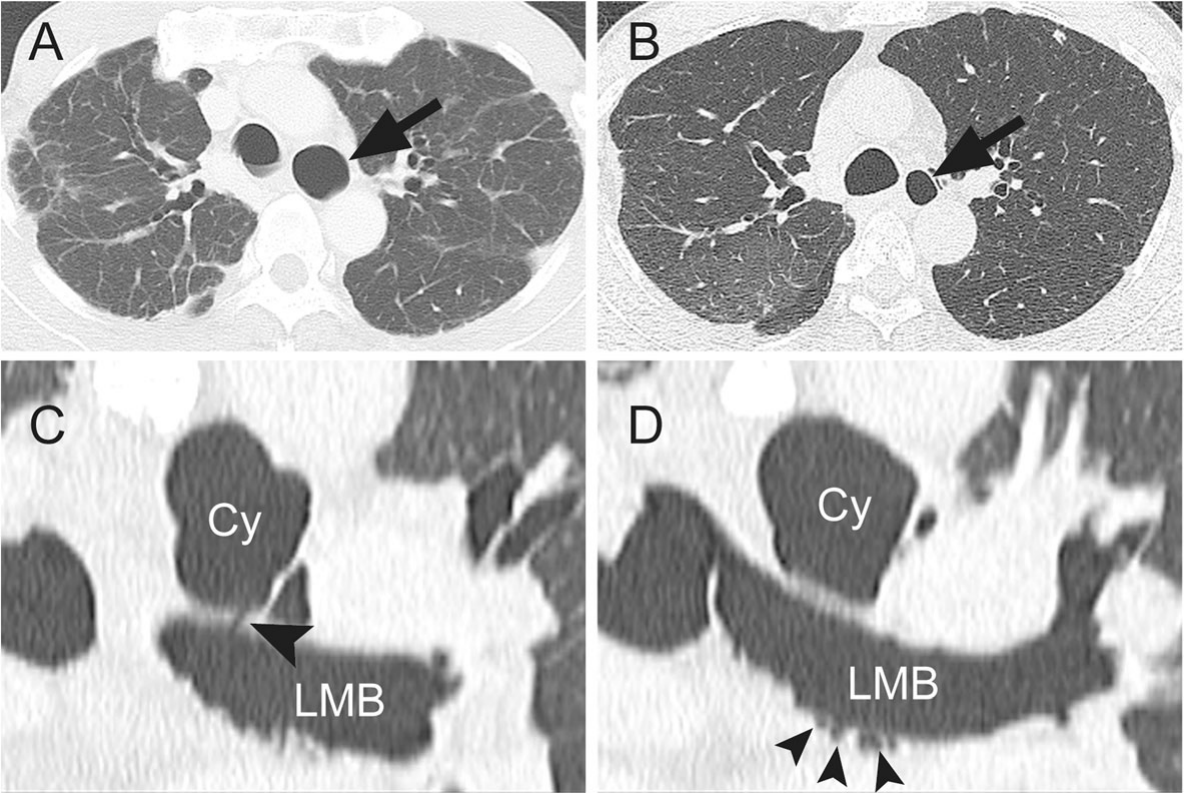
**a** Computed tomography (CT) showing a mediastinal air cyst (*arrow*) and fibrotic changes in the subpleural region. **b** CT obtained 4 years before the first visit to our hospital showing a smaller air cyst (*arrow*). **c** CT in the frontal plane showing a connection between the cyst (Cy) and the left main bronchus (LMB) (*arrowhead*). **d** CT in the frontal plane showing multiple extensions from the LMB (*arrowheads*)

**Fig. 2:**
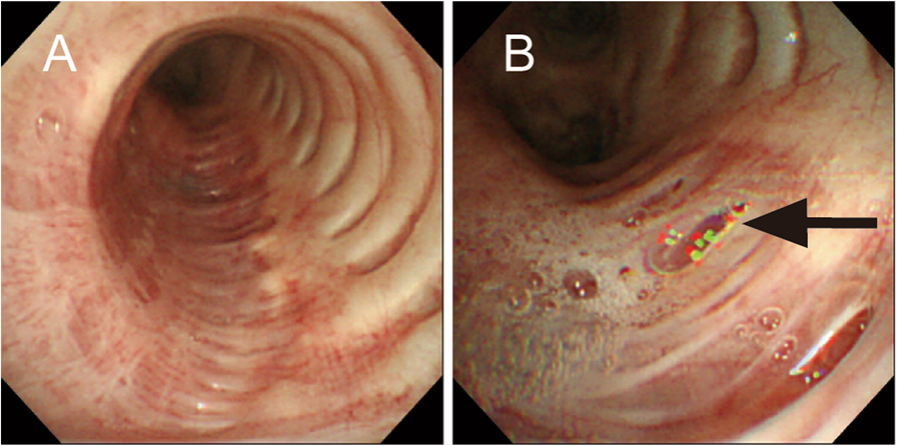
Bronchoscopic image showing the left main bronchus. **a** The reddish membranous portion is concave, thereby resulting in a round-shaped lumen. **b** Bubbling from slits or indentations of the bronchial mucosa (*arrow*)

**Fig. 3:**
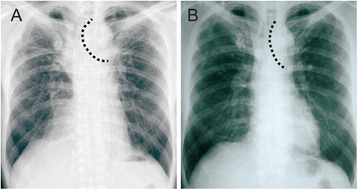
Chest radiograph taken at the **a** first visit and **b** 4 years before. The *dotted line* demarcates the left margin of the trachea



A 69-year-old man, who had regularly visited a chest physician for upper lobe-dominant pulmonary fibrosis and chronic cough, was referred to our hospital for the examination of an enlarged air-filled mediastinal cystic lesion detected using chest computed tomography (CT). The patient did not have any smoking history, worked at an iron cast manufacturing industry, and was prescribed antitussives for persistent cough by his physician. In laboratory studies upon admission, levels of white blood cells (4000/μL), lactate dehydrogenase (193 IU/L), C-reactive protein (0.04 mg/dL), and KL-6 (363 U/mL) were in the normal ranges. A pulmonary function test showed restrictive ventilatory impairment (vital capacity (VC) 2.16 L; %VC 60.7%; forced vital capacity (FVC) 2.33 L; forced expiratory volume in 1 s (FEV1) 2.25 L; FEV1/FVC 96.6%). CT showed a mediastinal air cyst (size 30 mm) adjacent to the LMB and dominant fibrous changes in the subpleural region in both upper lobes (Fig. 1a). A reconstructed frontal plane CT scan showed that the lumen of the cystic lesion was connected to that of the LMB through a thin tunnel, and that, many tiny air spaces extended from the LMB wall (Fig. 1c, d). Flexible bronchoscopy showed a round-shaped lumen of the LMB and bubbling from slits or indentations of the bronchial mucosa only in the LMB, but not in the right main bronchus or lobar bronchus (Fig. 2 and Additional file 1: Video 1). In comparison with the chest radiographs obtained during the first visit (Fig. 3a) and 4 years earlier (Fig. 3b), progression of volume loss in the right upper lobe was apparent. Compared with CT obtained 4 years earlier (Fig. 1b), the current CT (Fig. 1a) showed an enlargement of the air cyst over time.

**Additional file 1: Video 1.** Bronchoscopy of the left main bronchus showing bubbles from the slits or indentations of the mucosa (arrow). (7 MB)


**Fig. 4:**
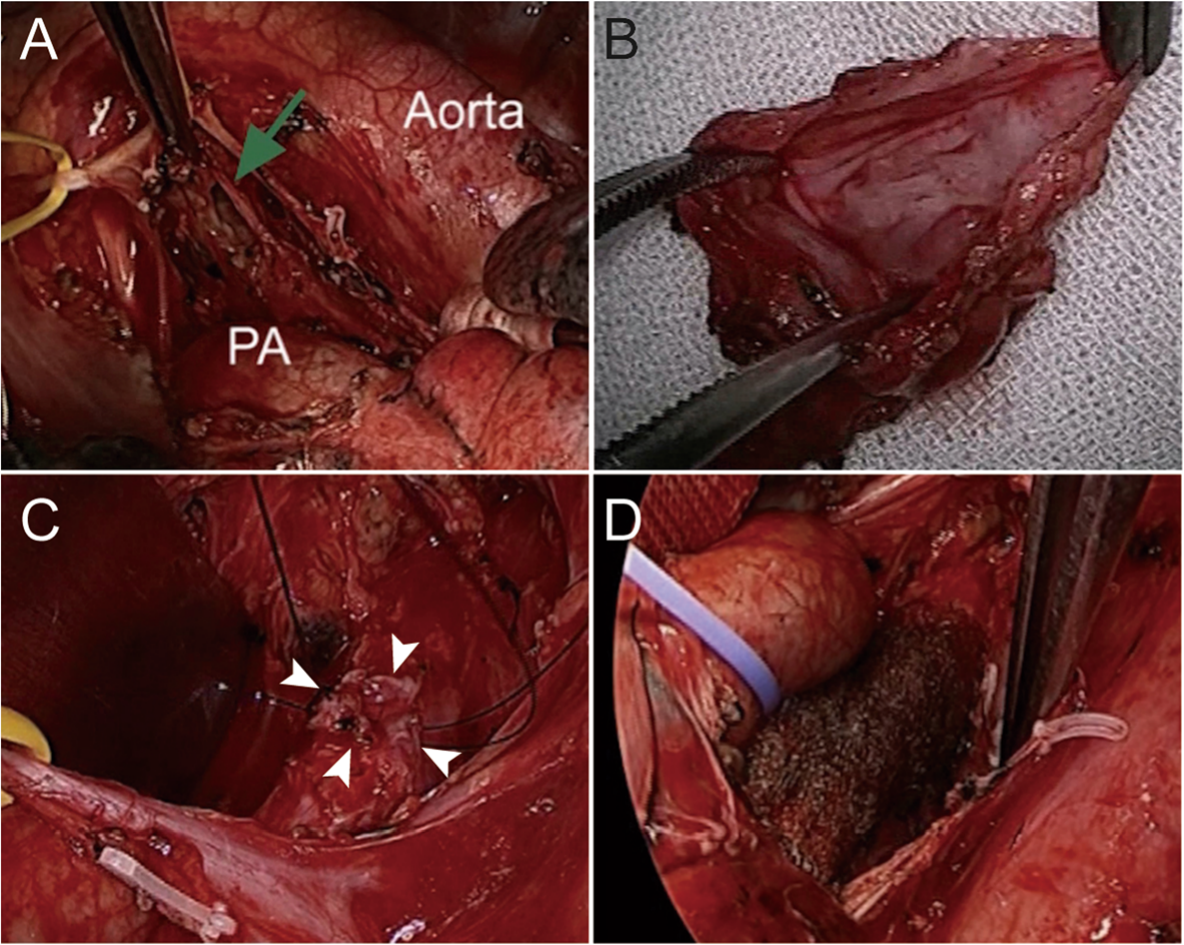
Intraoperative images showing **a** the opened lumen of the cystic lesion (*arrow*), **b** the resected specimen, **c** the placement of a Z-suture on the undissected portion of the cystic lesion on the left main bronchus (LMB) (*arrowheads*), and **d** the LMB covered with a polyglycolic acid sheet



For therapeutic diagnosis, we performed a removal of the cystic lesion under mini-thoracotomy using an 11-cm incision and two surgical ports (operative time 230 min; volume of blood loss 170 mL; Fig. 4 and Additional file 2: Video 2). Since it was difficult to identify the cystic lesion in the subaortic region (where it should exist) and fibrotic change of the region was apparent, we first identified the left recurrent nerve to avoid an accidental injury and subsequently attempted to dissect the cystic lesion. When we unintentionally made a hole in the wall of the lesion, we found the smooth, white-colored cystic lumen that enabled us to recognize the extent of the lesion. We nearly removed the entire cystic lesion that did not have adventitia. Since the cystic wall adjacent to the LMB could not be dissected due to its firm adhesion, an area of approximately 1 × 0.5 cm of the LMB was left with retained lesion tissue. When we completed a dissection of the whole LMB circumferentially, we did not recognize small diverticula that appeared on the preoperative CT, which indicates that the diverticula remained intact in the bronchial sheath.

**Additional file 2: Video 2.** Intraoperative video showing removal of the cystic lesion and the circumferential coverage of the left main bronchus with a polyglycolic acid sheet. (11 MB)


Air leakage from the LMB was seen only at the site where the cystic wall had not been dissected and was stopped by placing a Z-suture using 4–0 PDS II (Ethicon, Inc., Somerville, NJ). To reinforce the suture site and prevent another enlargement of tiny BDs, the patient underwent circumferential covering of the whole LMB with a polyglycolic acid (PGA) sheet. Finally, fibrin glue was applied on the PGA sheet.

The patient was discharged on postoperative day 11 without any adverse events.

**Fig. 5:**
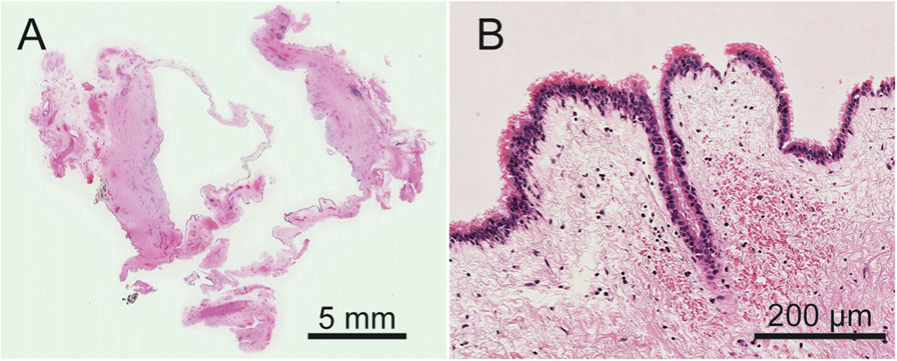
Pathological images of the resected cystic lesion. **a** Gross appearance indicating that the cystic wall consists of unstructured fibrous tissue without bronchial gland nor cartilage. **b** Microscopic image showing a ciliated epithelial lining

Upon pathological examination, the inner membrane of the lesion was covered with ciliated bronchial epithelial cells. The wall of the lesion consisted of unstructured fibrous tissue without bronchial gland tissue nor cartilage (Fig. 5). Follow-up bronchoscopy was performed 6 months postoperatively and indicated a disappearance of the bubbling as well as the resolution of the chronic cough to that of pre-procedural levels. The antitussive agent was discontinued postoperatively. Follow-up CT scans obtained at 1 year postoperatively showed no recurrence of air space or further development of BDs. Thereafter, he was monitored and treated by his prior chest physician. He died of chronic respiratory failure due to progression of upper lobe-dominant pulmonary fibrosis 20 months after the surgery.

## Discussion

Mediastinal air cysts in adults are extremely rare, and their differential diagnosis may include tracheocele (also known as paratracheal air cyst or tracheal diverticulum) [3], bronchogenic cyst [4], and bronchopulmonary foregut duplication cyst [5]. On the basis of our case, we recommend that BD should be added to this list.

The present case exhibited multiple tiny BDs in the LMB, and surgical findings confirmed the luminal connection between the LMB and cystic lesion. Pathological examination of the cyst revealed bronchial epithelial lining. Therefore, we diagnosed the air cyst as an enlarged BD. Regarding the etiology of BDs, chronic inflammation resulting from various causes and increased intrabronchial pressure due to coughing were associated with the development of BDs [1, 2]. In our case, BDs were detected only in the LMB. Additionally, BD enlargement coincided with a volume reduction of the right upper lobe, which resulted from the progression of upper lobe-dominant pulmonary fibrosis. Volume reduction of the right upper lobe resulted in the tracheal shift to the right that could induce the traction on the LMB (shown in Fig. 3). When considered together, we speculated that this mechanical traction of the LMB could be an initiating and/or an exacerbating factor of BDs. Furthermore, the absence of BDs in the trachea and lobar bronchus and more peripheral airway support the assumption that inhalation of particles or any underlying airway disease is less associated with the etiology of BDs in our case.

With respect to surgical indication, resection of an enlarged air cyst communicating with the airway would be warranted for pathological diagnosis and for eliminating the risk of future possible infection. Since we speculated the above-mentioned etiology, we covered the LMB with a PGA sheet to prevent enlargement of other BDs in the future. A 1-year postoperative workup using bronchoscopy and MDCT indicated neither the recurrence of air space nor any adverse event. Although the efficacy of this procedure is still inconclusive, we believe this is an option for treating diseases attributable to the mechanical vulnerability of the airway.

## Conclusions

This is the first case wherein bronchoscopic and surgical findings of bronchial diverticula and an enlarged bronchial diverticulum are reported. Mechanical traction and vulnerability of the airway might have had a role in inducing an enlargement of an air cyst in this case.

## Abbreviations

BD: Bronchial diverticula
CT: Computed tomography
LMB: Left main bronchus
MDCT: Multi-detector row computed tomography
PGA: Polyglycolic acid
